# Improved survival time trends in Hodgkin's lymphoma

**DOI:** 10.1002/cam4.655

**Published:** 2016-03-21

**Authors:** Matthew Koshy, Andrew Fairchild, Christina H. Son, Usama Mahmood

**Affiliations:** ^1^ Department of Radiation OncologyUniversity of Illinois at ChicagoChicagoIllinois60637; ^2^ Departments of Radiation and Cellular OncologyThe University of ChicagoChicagoIllinois60637; ^3^ Department of Radiation OncologyThe University of Texas MD Anderson Cancer CenterHoustonTexas

**Keywords:** Lymphoma, stage migration, time trends

## Abstract

There have been dramatic changes in the staging and treatment of Hodgkin's lymphoma (HL) over the past 30 years. We undertook this study to determine if a stage migration had occurred and also examined if treatment associated with later years has improved survival. Patients with stage I‐IV HL between 1983 and 2011 were selected from the Surveillance, Epidemiology, and End Results database. Multivariable analysis (MVA) was performed using Cox proportional hazards modeling. The study cohort included 35,680 patients. The stage breakdown in 1983 according to A and B symptoms was follows: 18%, 21%, 12%, and 5% for stage IA, IIA, IIIA, and IVA disease, respectively, and 6%, 11%, 12%, and 15% for stage IB, IIB, IIIB, and IVB disease. The stage breakdown in 2011 according to A and B symptoms was follows: 9%, 29%, 10%, and 6% for stage IA, IIA, IIIA, and IVA disease, respectively, and 4%, 16%, 12%, and 13% for stage IB, IIB, IIIB, and IVB disease. The median follow‐up for the entire cohort is 6.1 years. On MVA, the HR for mortality of patients diagnosed in 2006 was 0.60 (95% Confidence Interval (CI): 0.52–0.70) compared to 1983. For stage I and II patients diagnosed in 2006 the HR was 0.62 (95% CI: 0.44–0.87) and 0.40 (95% CI: 0.30–0.55), respectively, compared to patients diagnosed in 1983. For stage III and IV patients diagnosed in 2006 the HR was 0.72 (95% CI: 0.53–0.98) and 0.74 (95% CI: 0.56–0.99), respectively, compared to patients diagnosed in 1983. This is the first study to demonstrate a significant stage migration in early stage Hodgkin's lymphoma. Furthermore, these results demonstrate an improvement in survival over time for patients with Hodgkin's lymphoma which was particularly notable for those with early stage disease.

## Introduction

Over the last 75 years, advancements in the treatment of Hodgkin's lymphoma (HL) has changed its prognosis from being relatively incurable to one in which patients have a high likelihood of long‐term survival 1, 2. Refinements in the use of chemotherapy and radiation therapy has resulted in improved survival outcomes for patients with early‐ and late‐stage disease 3, 4, 5.

There have also been major advancements in the staging of HL. In 1980s lymphangiography and staging laparotomies were routinely performed to assess disease burden. These procedures have large been replaced due to advances in imaging techniques include widespread use of CT and PET scans to help guide treatment decisions 6. With the utilization of any new staging technique it is important to assess whether it causes an increase in patients who are upstaged or down‐staged compared to previously diagnosed patients. This presence of a “stage migration” in patients with HD has not been previously studied on a population wide level.

Also as improvements in survival was seen in the treatment of HL, concerns began to center around the long‐term toxicities of chemotherapy and radiation therapy 7, 8, 9. Recent trials have focused efforts on de‐intensifying treatment with chemotherapy and radiation therapy in order to mitigate the long‐term consequences from these treatments including the incidence of cardiopulmonary toxicities and the incidence of second malignances 10, 11. However, there are concerns that newer treatment paradigms which have de‐intensified treatments have resulted in worse survival outcomes on a population wide level 12, 13.

We undertook this study with two objectives, first to examine if changes in staging techniques associated with later years of diagnoses had resulted in a significant stage migration compared to patients diagnosed in an earlier time period. Furthermore, we sought to determine whether being diagnosed in a more recent year was associated with a survival improvement compared to patients treated in an earlier time period.

## Methods and Materials

All data are derived from the 1983–2013 database of the SEER Program of the United States National Cancer Institute, which includes data from 18 cancer registries (SEER‐18). The registry covers 26% of the US population. It contains information such as primary tumor site, age at diagnosis, gender, histologic type, stage, radiation status, B symptoms, and follow‐up. Information regarding local control, performance status, and specific radiation therapy technique (dose, fractionation, beam energy) is not available in the database. Furthermore, information on the receipt of chemotherapy is not listed in the database. The last potential date of diagnosis in our cohort was 2011. Patients with any other type of cancer prior to their HL diagnosis were excluded. Overall survival was the primary endpoint of our analysis, defined as the interval from the diagnosis to death from any cause. Cause‐specific death was not analyzed in this cohort because prior data has shown that the cause‐specific survival may be inaccurate in patients with localized disease or in those who develop secondary malignancies which is a known long‐term risk factor in patients with Hodgkin's lymphoma 14.

We included all patients with classical HL, stages I‐IV diagnosed from 1983 to 2011 and this resulted in 35,680 cases for analysis.

### Statistical analysis

Candidate variables included race, sex, age, histology, stage, and presence of B symptoms and year of diagnosis. All clinically relevant variables that are coded in the database were used in the analysis. The estimates of overall survival (OS) were calculated using the Kaplan–Meier method. The log–rank test was used to estimate whether differences were present in OS among these patients. All statistical tests were two‐sided and done at the 0.05 significance level.

All the candidate variables mentioned above were included as covariates in the multivariate analysis. Hazard ratios and the corresponding 95% confidence intervals were constructed in models adjusted for all listed covariates of interest. The data were analyzed using Statistical Analysis Systems, version 9.3 (SAS Institute, Cary, NC).

## Results

**Figure 1:**
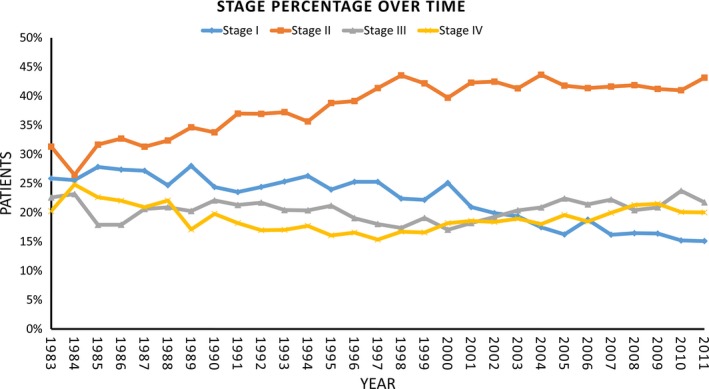
Stage Migration for Hodgkin's lymphoma from 1983 to 2011.

The study cohort included 35,680 patients. Table 1 lists the characteristics of our study population. The stage breakdown in 1983 according to A and B symptoms was follows: 18%, 21%, 12%, and 5% for stage IA, IIA, IIIA, and IVA disease, respectively, and 6%, 11%, 12%, and 15% for stage IB, IIB, IIIB, and IVB disease. The stage breakdown in 2011 according to A and B symptoms was follows: 9%, 29%, 10%, and 6% for stage IA, IIA, IIIA, and IVA disease, respectively, and 4%, 16%, 12%, and 13% for stage IB, IIB, IIIB, and IVB disease. An illustration of the changes in the ratio of stage I‐IV over time from 1983 to 2011 is shown in Figure 1.

**Table 1:** Patient and tumor characteristics

Characteristic (total population, *n* = 35,680)	Percentage (%)
Race	
White	84.8
Black	10.3
Other	4.9
Sex	
Male	54.1
Female	45.9
Lymphoma subtype	
Nodular sclerosis	63.7
Mixed cellularity	15.4
Lymphocyte rich	3.4
Classical Hodgkins NOS	15.9
Lymphocyte depleted	1.7
Ann arbor staging	
I	20.1
II	40.8
III	20.5
IV	18.6
B symptoms	
Yes	37.3
No	41.9
Unknown	20.8
Age	
≤ 30	41.4
> 30	58.6

**Figure 2:**
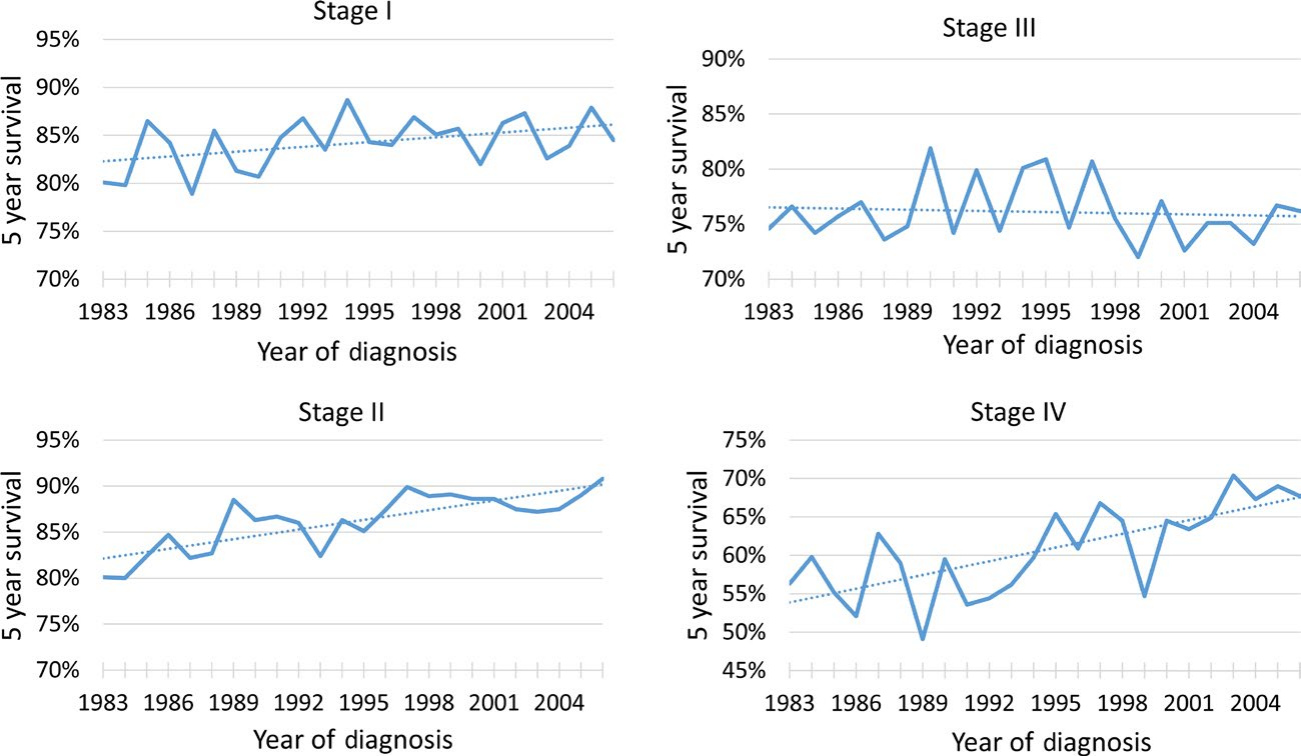
5 year Kaplan–Meier estimates of overall survival for stage I–IV patients with Hodgkin's lymphoma stratified by year of diagnosis.

The median follow‐up for the entire cohort is 6.1 years with an interquartile range of 2.3 years to 11.6 years. Five‐year OS (95% Confidence Interval (CI)) for the entire cohort continually improved through the years and was 73% (78.5–69.5%) versus 82% (84–80%) in 1983 versus 2006 (*P* < 0.0001), respectively. The changes in 5‐year OS of stages I‐IV from 1983 to 2006 is shown in Figure 2, A–D. For the years 1983 and 2006 the 5‐year OS (Number at Risk – NAR) for stage I changed from 80.1% (129) to 84.5% (284); stage II changed from 80.1% (156) to 90.8% (713); stage III changed from 74.6% (106) to 76.2% (299); and stage IV changed from 56.3% (56) to 67.7% (112).

Similarly, 15‐year OS for the cohort continually improved and was 58% versus 66% in 1983 versus 1996 (*P* < 0.0001). For the years 1983 and 2006 the 15‐year OS for stage I changed from 62.3% (NAR–96) to 67.2% (135); stage II changed from 64.1% (123) to 75.7% (240); stage III changed from 57.7% (82) to 58.0% (88); and stage IV changed from 45.2% (55) to 48.5% (62).

The relative 5 and 15‐year survival was calculated and was similar to the Kaplan–Meier calculated survival results. The 5‐year relative survival for the cohort in 1983 and 2006 was 77% (95% CI: 73–80%) versus 85.2% (95% CI: 83–87%). The 15‐year relative survival for the cohort in 1983 and 1996 was 63% (95% CI: 59–67%) versus 72% (95% CI: 69–76%).

**Figure 3:**
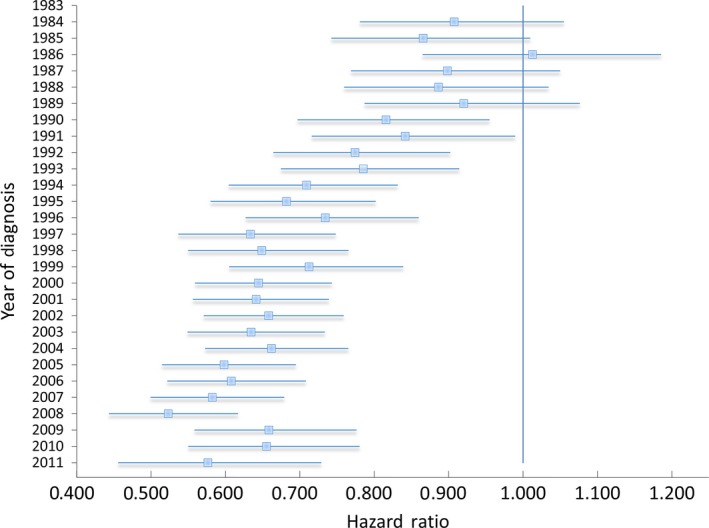
Multivariate Analysis of Survival for Hodgkin's Lymphoma, Stages I‐IV, with Forest Plot Depicting Adjusted Hazard Ratio (95% Confidence Interval) of Survival Stratified by Year of Diagnosis Compared to 1983 as the referent group.

The multivariate analysis of survival for all Hodgkin's lymphoma patients is shown in Table 2 and the corresponding forest plot from the model demonstrating improved survival with later years of diagnosis is shown in Figure 3. On MVA, in patients with a minimum of 5 years of follow‐up, the HR for mortality of patients diagnosed in 2006 was 0.60 (95% Confidence Interval (CI): 0.52–0.70) compared to 1983. We performed a sensitivity analysis which included the SEER registry region as a separate variable in the MVA and found it did not significantly affect the hazard ratios of the other covariates in the model and also that no region was associated with a statistically different hazard ratio.

**Table 2:** Multivariate analysis for overall survival

Covariate	Hazard Ratio	95% Confidence Interval	*P* ‐value
Race			
White	1.0 (ref)		*P* < 0.001
Black	1.16	1.08–1.23	
Other	1.08	0.96–1.23	
Sex			
Male	1.0 (ref)		*P* < 0.001
Female	0.90	0.86–0.95	
Lymphoma subtype			
Nodular sclerosis	1.0 (ref)		*P* < 0.001
Mixed cellularity	1.56	1.48–1.64	
Lymphocyte rich	1.23	1.10–1.38	
Classical Hodgkins NOS	1.82	1.72–1.93	
Lymphocyte depleted	2.68	2.40–2.99	
Ann arbor staging			
I	1.0 (ref)		*P* < 0.001
II	0.95	0.89–1.00	
III	1.44	1.35–1.54	
IV	1.86	1.75–1.98	
B symptoms			
Yes	1.0 (ref)		*P* < 0.001
No	0.67	0.64–0.70	
Unknown	0.90	0.85–0.94	
Age			
≤ 30	1.0 (ref)		*P* < 0.001
> 30	3.61	3.42–3.82	
Year of diagnosis			
1983	1.0 (ref)		*P* < 0.001
1984	0.91	0.78–1.05	
1985	0.87	0.74–1.01	
1986	1.01	0.86–1.19	
1987	0.90	0.77–1.05	
1988	0.89	0.76–1.03	
1989	0.92	0.79–1.08	
1990	0.82	0.7–0.95	
1991	0.84	0.72–0.99	
1992	0.77	0.66–0.9	
1993	0.79	0.67–0.91	
1994	0.71	0.6–0.83	
1995	0.68	0.58–0.8	
1996	0.73	0.63–0.86	
1997	0.63	0.54–0.75	
1998	0.65	0.55–0.77	
1999	0.71	0.6–0.84	
2000	0.64	0.56–0.74	
2001	0.64	0.56–0.74	
2002	0.66	0.57–0.76	
2003	0.63	0.55–0.73	
2004	0.66	0.57–0.77	
2005	0.60	0.51–0.69	
2006	0.61	0.52–0.71	
2007	0.58	0.50–0.68	
2008	0.52	0.44–0.62	
2009	0.66	0.56–0.78	
2010	0.66	0.55–0.78	
2011	0.58	0.46–0.73	

**Figure 4:**
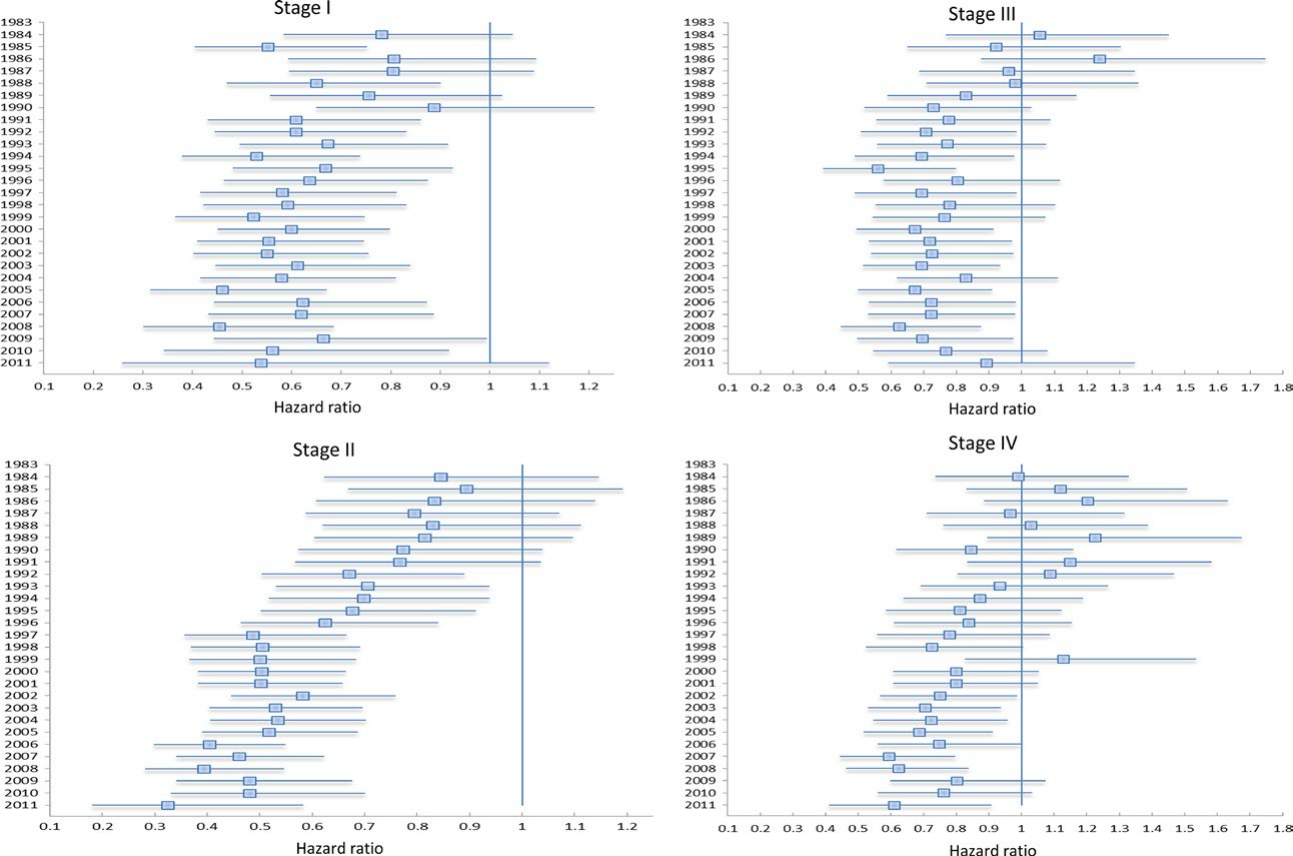
Multivariate Analysis of Survival for Hodgkin's Lymphoma, Stratified by Stage, with Forest Plot Depicting Adjusted Hazard Ratio (95% Confidence Interval) of Survival by Year of Diagnosis Compared to 1983 as the referent group.

To further examine survival trends by year and stage, a separate MVA was performed within each stage grouping. The corresponding forest plots from the models demonstrating the HR trends by year of diagnosis are shown in Figure 4. For stage I and II patients diagnosed in 2006 the HR was 0.62 (95% CI: 0.44–0.87) and 0.40 (95% CI: .30–0.55), respectively, compared to patients diagnosed in 1983. For stage III and IV patients diagnosed in 2006 the HR was 0.72 (95% CI: 0.53–0.98) and 0.74 (95% CI: 0.56–0.99), respectively, compared to patients diagnosed in 1983.

## Discussion

Our analysis of the most recent SEER data demonstrates a stage migration with a higher percentage of patients diagnosed with stage II disease. Also, there was a gradual improvement in OS over the time period studied. Early‐stage disease showed marked improvement in both 5‐ and 15‐year OS, whereas advanced stage disease showed improved 5‐year OS without any major difference in long‐term survival improvement.

Between 1983 and 2011, our analysis demonstrated a decrease in the diagnosis of Stage I HL (−21%), with an almost equal and opposite increase in Stage II (+21%), whereas the incidence of Stages III and IV disease remained relatively constant. After 1997 the increase in stage II disease stabilized. There have been considerable changes to the staging of Hodgkin's lymphoma which occurred throughout this time which may have contributed to this finding. Initially, staging techniques in the 1960s utilized lymphangiography which was later replaced by the more sensitive staging laparotomy 15. In the 1980s, advancements in imaging techniques utilizing computed tomography came into widespread use. This was later followed by further advancements in imaging technology including gallium scans and finally the widespread use of PET scans which provided a more sensitive assessment of initial disease burden 16, 17, 18, 19, 20. The widespread adoption of radiographic imaging in the mid 1990s may be the reason why there have been no dramatic changes in stage migration since 1997 according to Figure 1. The improvement in survival in stage I‐II disease can partly be attributed to this stage migration, that occurred previous to the mid 1990s, where previously staged I patients with a good prognosis were now reclassified as stage II patients thereby raising the survival in both groups, otherwise known as the “Will Rogers” phenomenon 21.

Our study found continued improvements in overall survival over the time period studied, especially in 5‐year overall survival for patients with early‐stage disease. It was encouraging to note that advances in treatment techniques including the utilization of multi‐agent chemotherapy, and smaller radiation fields and doses, and improved salvage therapies have all led to population wide benefits in survival in Hodgkin's lymphoma 22, 23. However, this study did demonstrate very limited improvements in 15‐year overall survival for patients with stage III‐IV disease. This is likely due to long‐term effects of the treatments received and further efforts are needed minimize treatment‐related mortalities and complications. Enrollment of Hodgkin's lymphoma patients in survivorship clinics is paramount to combat the increased treatment related long‐term mortality seen in this population 24, 25, 26.

The multivariate analysis revealed a continued reduction in mortality for all stages of Hodgkin's lymphoma from 1983 to 2011. On further subset analysis, it was revealed that the major reduction in mortality was seen in early‐stage patients. One trend that requires further consideration is the worsening trend mortality in patients with stage III disease in later years which saw a continued nonsignificant increase in HR from 0.63 in 2008 to 0.89 in 2011. Further research will be needed to determine if this trend reaches statistical significance in later years and if this finding is related to recent changes in chemotherapy regimens or changes in the utilization of radiation therapy over the time period studied. Also, consistent with previous studies, blacks and males had slightly worse survival outcomes on multivariate analysis 27.

This study has the limitations of being a retrospective review. Information regarding radiotherapy specifics like field size, dose, fractionation, or beam energy, as well as chemotherapy‐specific like combination regimen, dose, and cycles, imaging modalities and the use of salvage therapies is not available. Also, the presence of B symptoms was not coded in over 20% of the cohort. Furthermore, other disease features including comorbidities, cause of death, and factors from the Hasenclever international prognostic score such as albumin and hemoglobin levels, and presence of leukocystosis or lymphopenia remain unknown and could have significantly affected the multivariate analysis 28. Also, we included patients diagnosed with Hodgkin's lymphoma in earlier treatment era's where modern immunohistochemistry diagnostic techniques were not in practice.

In conclusion, this is the first study to reveal a significant stage migration in early stage Hodgkin disease with a larger proportion now presenting with stage II compared to stage I disease. Furthermore, these results demonstrate a remarkable improvement in mortality for all stages of Hodgkin lymphoma which was particularly significant for those with early stage disease.

## Conflicts of Interest

None declared.
